# Insulin-stimulated lipid accumulation is inhibited by ROS-scavenging chemicals, but not by the Drp1 inhibitor Mdivi-1

**DOI:** 10.1371/journal.pone.0185764

**Published:** 2017-10-02

**Authors:** Jung-Hak Kim, Sun-Ji Park, Bokyung Kim, Young-Geun Choe, Dong-Seok Lee

**Affiliations:** School of Life Sciences, BK21 Plus KNU Creative BioResearch Group, Kyungpook National University, Daegu, Republic of Korea; Universidad Pablo de Olavide, SPAIN

## Abstract

Adipocyte differentiation is regulated by intracellular reactive oxygen species (ROS) generation and mitochondrial fission and fusion processes. However, the correlation between intracellular ROS generation and mitochondrial remodeling during adipocyte differentiation is still unknown. Here, we investigated the effect on adipocyte differentiation of 3T3-L1 cells of intracellular ROS inhibition using N-acetyl cysteine (Nac) and Mito-TEMPO and of mitochondrial fission inhibition using Mdivi-1. Differentiated 3T3-L1 adipocytes displayed an increase in mitochondrial fission, ROS generation, and the expression of adipogenic and mitochondrial dynamics-related proteins. ROS scavenger (Nac or Mito-TEMPO) treatment inhibited ROS production, lipid accumulation, the expression of adipogenic and mitochondrial dynamics-related proteins, and mitochondrial fission during adipogenesis of 3T3-L1 cells. On the other hand, treatment with the mitochondrial fission inhibitor Mdivi-1 inhibited mitochondrial fission but did not inhibit ROS production, lipid accumulation, or the expression of adipogenic and mitochondrial dynamics-related proteins, with the exception of phosphorylated Drp1 (Ser616), in differentiated 3T3-L1 adipocytes. The inhibition of mitochondrial fission did not affect adipocyte differentiation, while intracellular ROS production decreased in parallel with inhibition of adipocyte differentiation. Therefore, our results indicated that ROS are an essential regulator of adipocyte differentiation in 3T3-L1 cells.

## Introduction

Obesity increases the number (hyperplasia) and size (hypertrophy) of adipocyte cells [1, 2]. It can lead to many health problems, such as type 2 diabetes, insulin resistance, coronary heart disease, and cancer [3]. Adipocytes are responsible for lipid uptake, synthesis, and storage in the form of triglyceride (TG). Abnormal accumulation of stored TG in adipocytes causes obesity [4]. For this reason, many researchers have intensively studied the cellular and molecular mechanisms of adipocyte differentiation.

Adipogenesis is a cellular differentiation process by which preadipocytes become mature adipocytes. Adipocyte differentiation is a complex developmental process accompanied by coordinated changes in cell morphology, hormone sensitivity, and gene expression [5]. The adipogenic hormone insulin triggers the induction of a series of transcription factors governing adipocyte differentiation [6, 7]. Insulin-mediated activation of protein kinase B (AKT) promotes glucose uptake in adipocytes by leading vesicle of glucose transporter 4 (GLUT4) to moving into the plasma membrane [8–10]. In addition, activation of AKT also enhances the expression of peroxisome proliferator-activated receptor γ (PPARγ) and CCAAT/enhancer-binding protein α (C/EBPα) by mediating insulin signals. PPARγ and C/EBPα are key adipogenic transcription factors that collaborate to elevate expression of adipocyte-specific genes, such as GLUT4 and fatty acid-binding protein 4 (FABP4, also known as aP2) [11, 12]. Thus, these genes are the key factors for regulating the adipocyte differentiation program.

Reactive oxygen species (ROS) have been introduced to be mainly produced by NADPH oxidase 4 (Nox4) or mitochondrial enzymes after induction of adipocyte differentiation [12, 13]. Several researchers have considered that intracellular ROS are necessary for adipocyte differentiation [13, 14]. Intracellular ROS generation through Nox4 occurs during the early stages of insulin-mediated adipogenesis, which enhances insulin signaling transduction [15]. ROS generated at mitochondrial complex III are required to initiate adipocyte differentiation through the induction of PPARγ transcriptional machinery [16]. In addition, ROS promote adipocyte differentiation. Both ROS generation and adipocyte differentiation are lowered by Nox4 knockdown and mitochondria specific antioxidants in mesenchymal stem cells [13, 17]. Adipogenesis is accelerated with increased expression of PPARγ in 3T3-L1 cells treated with hydrogen peroxide [18]. Therefore, ROS are required for the process of adipocyte differentiation.

Differentiation is a highly energy-demanding process [19]. Cellular bioenergetic function is regulated by mitochondrial dynamics, a concept that encompasses the regulation of mitochondrial architecture mediated by movement, fusion, and fission. The fusion of mitochondrial compartments allows the generation of interconnected mitochondria, whereas fission produces numerous mitochondrial fragments [20]. Mitochondrial fusion and fission processes play an important role in energy metabolism, cell differentiation, and apoptotic cell death [21]. Interestingly, mitochondrial fusion and fission have a direct influence on TG accumulation in the adipocyte. Differentiated 3T3-L1 adipocytes displayed fragmented and punctate mitochondria surrounding lipid droplets, and an increase in the expression of the mitochondrial fission protein dynamin-related protein 1 (Drp1) and the mitochondrial fusion protein mitofusion 2 (Mfn2) [22]. Meanwhile, the induction of mitochondrial fusion by silencing of Drp1 and fission 1 homolog protein (Fis1) causes a decrease in cellular TG content, while the induction of mitochondrial fission by silencing of Mfn2 and optic atrophy-1 (OPA1) causes an increase in cellular TG content in 3T3-L1 cells [23]. Taken together, these studies revealed that intracellular ROS generation, as well as mitochondrial dynamics regulation, contributes to the control of adipocyte differentiation and lipid accumulation. However, the correlation between insulin-induced ROS generation and mitochondrial remodeling during adipocyte differentiation is not fully understood.

Here, we examined the effect of Mdivi-1, an inhibitor of the mitochondrial fission protein Drp1, on insulin-induced lipid accumulation, adipogenic gene expression, and intracellular ROS generation during differentiation of 3T3-L1 cells. In addition, we investigated the effect of the broad ROS scavenger N-acetyl cysteine (Nac) and the mitochondria-targeted ROS scavenger Mito-TEMPO on mitochondrial morphology and the expression of mitochondrial dynamics-related and adipogenic proteins.

## Materials and methods

### Cell culture, differentiation, and treatments

We purchased 3T3-L1 preadipocytes from the American Type Culture Collection (Manassas, VA, USA). Cells were cultured at 37°C/5% CO_2_ in Dulbecco’s modified Eagle’s medium (DMEM) containing 4500 mg/L glucose (Welgene, Korea), supplemented with 1% penicillin/streptomycin (Welgene) and 10% bovine calf serum (Gibco, New Zealand). Cultures were allowed to grow to confluency; after 48 h, cells were treated with a hormone mixture (MDI) and 10% fetal bovine serum (FBS; Gibco) for 2 days. The MDI mixture was composed of 0.5 mM 3-isobutyl-1-methylxanthine, 1 μM dexamethasone, and 1 μg/mL insulin (Sigma-Aldrich, MO, USA). Following the initiation of differentiation, the culture medium was replenished every 2 days with DMEM (10% FBS) supplemented only with 1 μg/mL insulin for 6 days. We used 3T3-L1 cells until they reached passage 10. All experiments were performed five times, and each experiment was independently analyzed.

N-acetyl-L-cysteine (Nac; Sigma) or Mdivi-1 (Sigma) or Mito-TEMPO (Enzo Life Sciences, NY, USA) treatments were initiated at 48 h after MDI treatment. 3T3-L1 cells were pretreated with Nac, or Mdivi-1 or Mito-Tempo for 30 min and then treated with 1 μg/mL insulin.

### Protein extraction and western blotting

Proteins were extracted from cells using PRO-PREP Protein Extraction Solution (iNtRON, Korea). Cell extracts were separated by 12% SDS-PAGE and transferred onto nitrocellulose membranes (Pall, FL, USA). Membranes were incubated with antibodies against PPARγ (sc-271392, mouse monoclonal antibody, 1:1000, Santa Cruz, Dallas, TX, USA), C/EBPα (sc-61, rabbit polyclonal antibody, 1:1000 Santa Cruz), Drp1 (sc-32898, rabbit polyclonal antibody, 1:2000 Santa Cruz), Mfn1 (sc-50330, rabbit polyclonal antibody, 1:2000 Santa Cruz), Mfn2 (sc-50331, rabbit polyclonal antibody, 1:2000 Santa Cruz), β-actin (sc-47778, rabbit polyclonal antibody, 1:5000 Santa Cruz), AKT (#9272, rabbit polyclonal antiboy, 1:1000, Cell Signaling, Danvers, MA, USA), phosphorylated (p)-AKT (Ser473) (#9271, rabbit polyclonal antiboy, 1:1000, Cell Signaling), Glut4 (#2213, rabbit polyclonal antiboy, 1:1000, Cell Signaling), FABP4 (aP2) (#2120, rabbit polyclonal antiboy, 1:1000, Cell Signaling), p-Drp1 (Ser616) (#3455, rabbit polyclonal antiboy, 1:1000, Cell Signaling), p-Drp1 (Ser637) (#4867, rabbit polyclonal antiboy, 1:1000, Cell Signaling), Opa1 (612607, mouse monoclonal antibody, 1:5000, BD Biosciences, Franklin Lakes, NJ, USA), superoxide dismutase (SOD)1 (LF-PA0013, rabbit polyclonal antibody, 1:2000, AbFrontier, Seoul, Korea), SOD2 (LF-MA0030, mouse monoclonal antibody, 1:2000, AbFrontier, Seoul, Korea), peroxiredoxin (Prx)1 (LF-PA0095, rabbit polyclonal antibody, 1:2000, AbFrontier, Seoul, Korea), Prx2 (LF-MA0144, mouse monoclonal antibody, 1:2000, AbFrontier, Seoul, Korea), Prx3 (LF-MA0044, mouse monoclonal antibody, 1:2000, AbFrontier, Seoul, Korea), Prx4 (LF-PA0009, rabbit polyclonal antibody, 1:2000, AbFrontier, Seoul, Korea), Prx5 (LF-PA0210, rabbit polyclonal antibody, 1:2000, AbFrontier, Seoul, Korea), and Prx6 (LF-PA0011, rabbit polyclonal antibody, 1:2000, AbFrontier, Seoul, Korea). We used horseradish peroxidase-conjugated anti-mouse IgGs (31439, 1:5000, goat polyclonal antibody, Thermo Fisher Scientific, Waltham, MA, USA), and horseradish peroxidase-conjugated anti-rabbit IgGs (31460, 1:5000, goat polyclonal antibody, Thermo Fisher Scientific) as secondary antibodies. Signals were visualized using Clarity Western ECL Substrate (Bio-Rad, Hercules, CA, USA). Band intensities were analyzed using Multi Gauge version 3.0 (Fujifilm, Tokyo, Japan).

### Lentivirus generation and establishment of a stable cell line

The DsRed2-Mito gene was obtained from the pDsRed2-Mito vector (Clontech, CA, USA), and the coding sequence was amplified by PCR with LA Taq polymerase (Takara, Shiga, Japan). Amplified DsRed2-Mito was cloned into the entry pCR8/GW/TOPO vector (Invitrogen) to generate expression clones by performing LR recombination between the entry vector and the destination vector, pLenti6.3/V5-DEST (Invitrogen). All three vectors, pLenti6.3-DsRed2-Mito, psPAX2 packaging, and pMD.2G enveloping vectors, were transfected into HEK293FT cells using Lipofectamine 2000 (Invitrogen). After 12 h of transfection, the medium was exchanged for fresh medium. Then, 48 to 72 h after transfection, the lentivirus-containing medium was harvested and purified with a 0.45-μm filter (Sartorius, Gottingen, Germany).

pLenti6.3-DsRed2-Mito stable cell lines were generated by infecting 3T3-L1 cells with lentiviral vectors. During infection, polybrene (Sigma), which increases the transfection efficiency, was added at 8 μg/mL, and the cells were cultured for 72 h. Single cells with high and stable RFP expression were selected and cloned by sorting using a FACSAria II cell sorter. The sorter was equipped with a laser turned to 488 nm. The RFP fluorescence from the cell was measured and detected by PE (563~589 nm), and the collected data was quantified and analyzed using FACSDiva version 6.1.3. The sorter then applied a charge to the droplet containing the cell to sort into a collection tube.

### Mitochondrial imaging

DsRed2-Mito-expressing 3T3-L1 cells were seeded on 0.1% poly-D-lysine-coated round coverslips. After MDI treatment, cells were treated with 1 μg/mL insulin for 6 days, with or without Nac, or Mdivi-1, or Mito-Tempo. The cells were then washed twice with PBS and fixed with 4% paraformaldehyde in PBS for 3 h. Images were obtained using a LSM-710 confocal microscope (Carl Zeiss, Germany) and processed using Zeiss LSM Image examiner, ZEN 2009 light edition (Carl Zeiss,). Mitochondrial length was measured using ImageJ software (NIH, MD, USA) and calculated using more than 50 mitochondria per cell from 20 cells. Mitochondria were divided into different categories based on their length, i.e., less than 1, 1–3, and more than 3 μm.

### Detection of intracellular and mitochondrial ROS

Generation of intracellular and mitochondrial ROS was detected using 2,7-dichlorofluorescein diacetate (CM-H_2_DCF-DA; Invitrogen, CA, USA) and MitoSOX (Invitrogen). At 8 days after the initiation of differentiation, cells were treated with 1 μM CM-H_2_DCF-DA or 2 μM MitoSOX for 20 min at 37°C. Cells were then washed with PBS and examined under a fluorescence microscope (Leica Microsystems, Germany).

### Oil Red O staining

Lipid accumulation was detected using Oil Red O (Sigma-Aldrich). At 8 days after the initiation of differentiation, cells were fixed with 4% paraformaldehyde (Sigma-Aldrich) and then stained with 0.35% Oil Red O solution for 3 h at 25°C. Cells were washed three times using double-distilled water. The red-stained lipid droplets were observed under a light microscope. To quantify lipid accumulation, the Oil Red O was eluted by adding 100% isopropanol and then absorbance was measured at 495 nm.

### Statistical analysis

Values are presented as the means ± SEM of five or more independent experiments. For group comparisons, one-way analysis of variance followed by Dunnett’s multiple comparison tests were performed with GraphPad Prism version 4.0 (GraphPad Software, CA, USA). A p value less than 0.05 was considered statistically significant.

## Results

### Expression of adipogenic, mitochondrial dynamics, and antioxidant gene proteins induced during adipocyte differentiation

**Fig 1:**
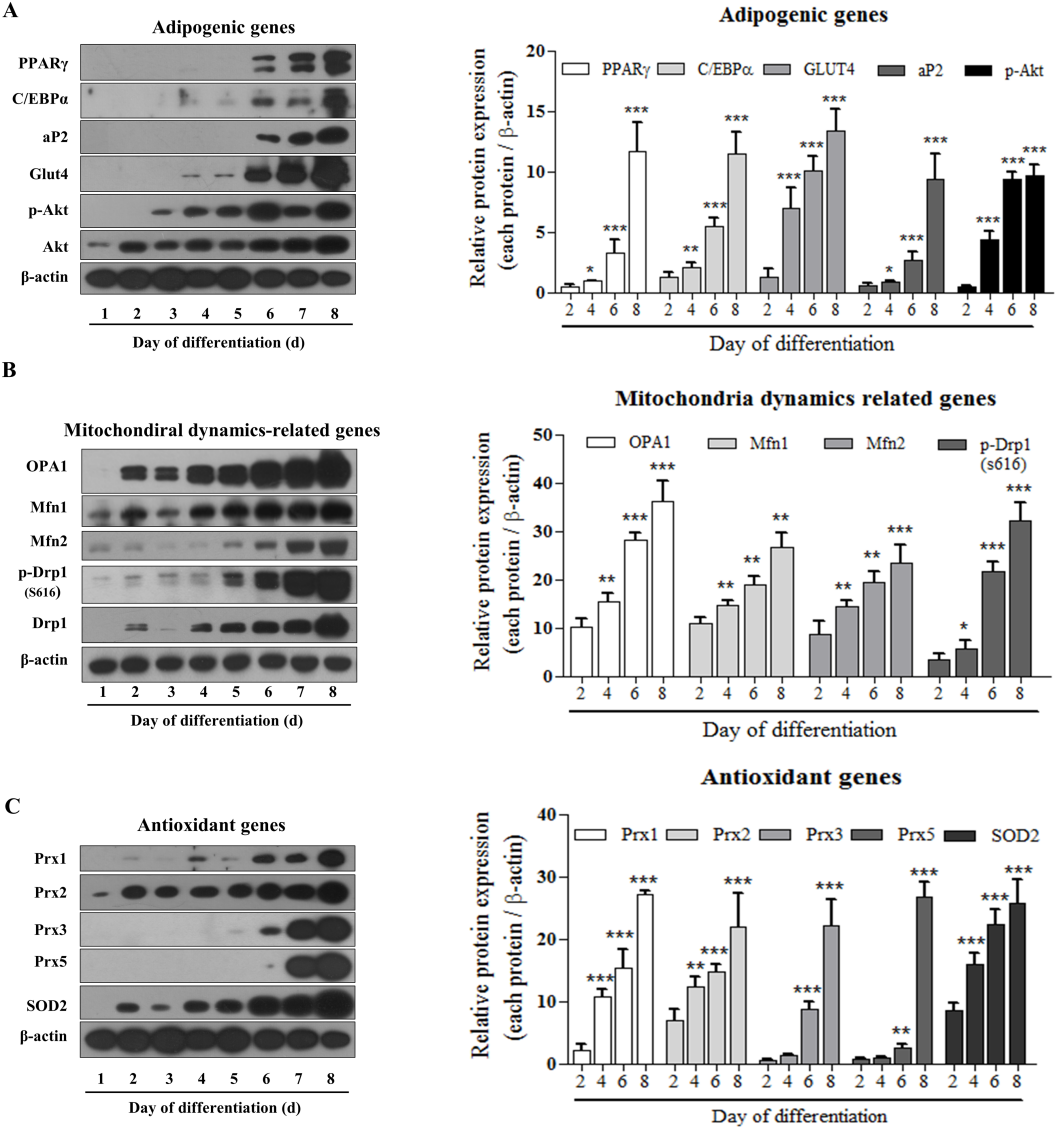
Changes in expression levels of adipogenic, antioxidant, and mitochondrial dynamics-related genes during adipogenesis of 3T3-L1 cells. 3T3-L1 cells were harvested daily for 8 days during differentiation of pre-adipocytes into adipocytes following MDI treatment. Cells were lysed, and protein extracts were subjected to western blot analysis with the indicated antibodies: adipogenic genes (PPARγ, C/EBPα, p-AKT, AKT, GLUT4, and aP2), mitochondrial dynamics-related genes (OPA1, Mfn1, Mfn2, p-Drp1 (Ser616), and Drp1), and antioxidant genes (Prx1, Prx2, Prx3, Prx5, and SOD2). Data in the bar graphs represent the means ± SEM of three independent experiments. *p<0.05, **p < 0.01, ***p < 0.001, compared with early differentiated adipocytes (day 1).

Insulin treatment led 3T3-L1 to differentiation into mature adipocyte. We investigated the altered expression of adipogenic, mitochondrial dynamics, and antioxidant genes during adipocyte differentiation. First, we examined the expression of adipogenic genes at several time points during adipocyte differentiation. Expression of the adipogenic markers PPARγ, C/EBPα, aP2, and GLUT4 was significantly increased at day 8 compared to day 2, and phosphorylated AKT expression levels also gradually increased during adipocyte differentiation (Fig 1A). Next, we investigated mitochondrial dynamics-related gene expression at several time points during adipogenesis. Expression levels of mitochondrial fusion related proteins, such as OPA1, Mfn1, and Mfn2, gradually increased, as did that of the mitochondrial fission related protein, Drp1, and levels of phosphorylated serine 616 Drp1 also gradually increased during adipocyte differentiation (Fig 1B). According to our previous study, insulin treatment elevated expression of antioxidant enzymes in mature adipocytes [24]. We confirmed the altered expression of antioxidant enzyme levels during adipocyte differentiation. Levels of the antioxidant proteins Prx1, Prx2, Prx3, Prx5, and SOD2 gradually increased, and were especially high after 8 days (Fig 1C). Therefore, expression of adipogenic, mitochondrial dynamics, and antioxidant gene proteins was increased during adipocyte differentiation.

### Insulin-stimulated lipid accumulation is inhibited by antioxidants, but not by the Drp1 inhibitor, Mdivi-1

**Fig 2:**
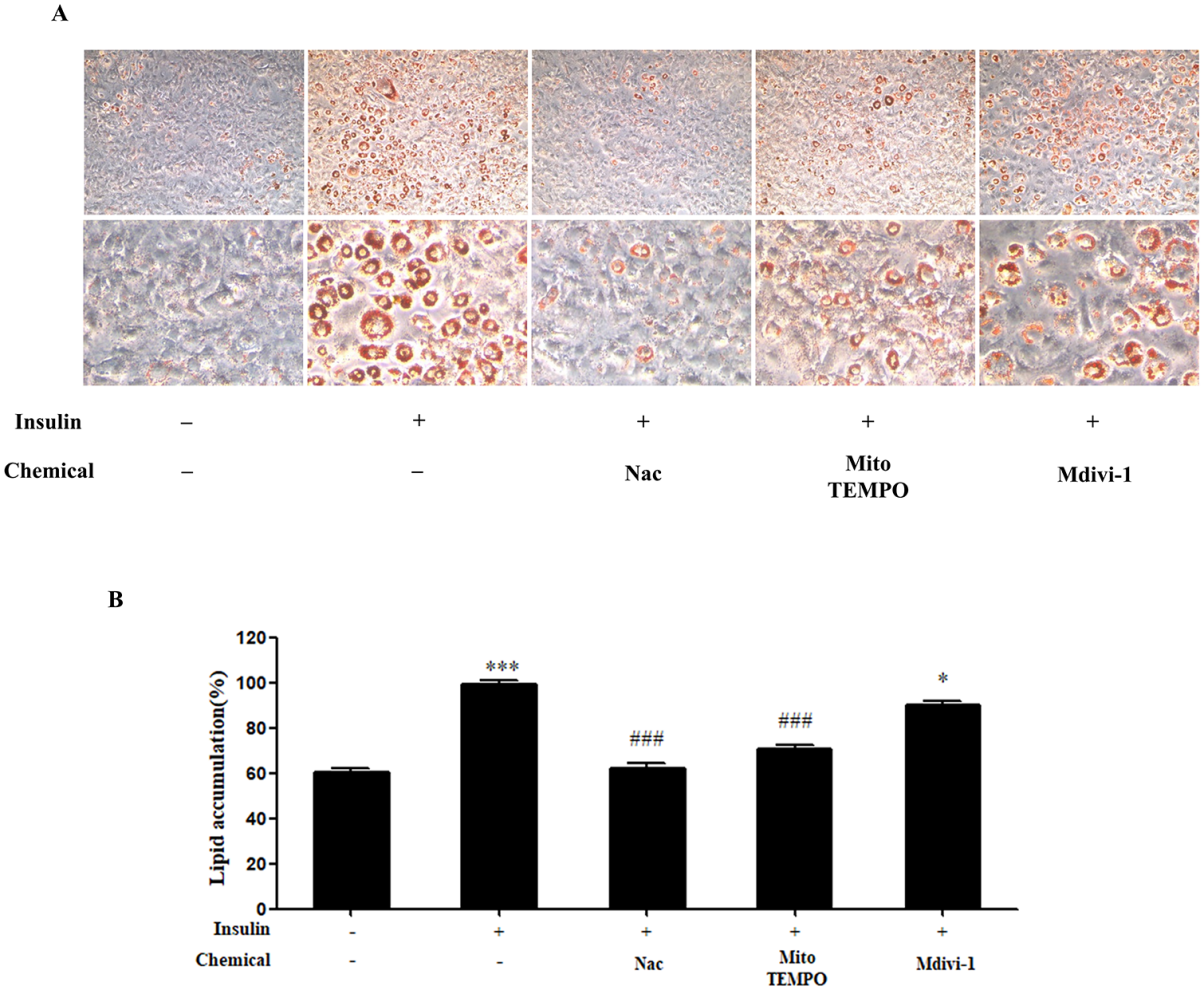
Effect of N-acetyl cysteine (Nac), Mito-TEMPO, and Mdivi-1 on lipid accumulation in 3T3-L1 cells during adipogenesis. 3T3-L1 adipocytes were pretreated with 10 mM Nac, 200 μM Mito-TEMPO, or 50 μM Mdivi-1 for 30 min and then with insulin during adipogenesis. (A) Accumulated lipid was stained with Oil Red O reagent and quantified by absorbance at 490 nm (B). Data in the bar graph represent the means ± SEM of three independent experiments. *p < 0.05, ***p < 0.001, compared with untreated or insulin-treated cells. ^###^p<0.001, compared with insulin-treated cells.

Next, we investigated whether antioxidants or Mdivi-1 could reduce lipid accumulation in mature adipocytes. We added insulin to 3T3-L1 cells pretreated with Nac, Mito-TEMPO, or Mdivi-1 during adipocyte differentiation. As shown in our oil red o staining results, lipid was significantly more accumulated in insulin-treated than in non-treated cells (Fig 2A and 2B). Treatment with Nac or Mito-TEMPO and insulin resulted in a significant decrease in lipid accumulation compared with insulin treatment alone (Fig 2A and 2B). However, insulin-induced lipid accumulation was not reduced by Mdivi-1 (Fig 2A and 2B). Our results show that antioxidant treatment results in inhibited insulin-stimulated lipid accumulation; however, Mdivi-1 did not inhibit lipid accumulation.

### Expression levels of insulin-induced adipogenic related genes are inhibited by antioxidants, but not by the Drp1 inhibitor, Mdivi-1

**Fig 3:**
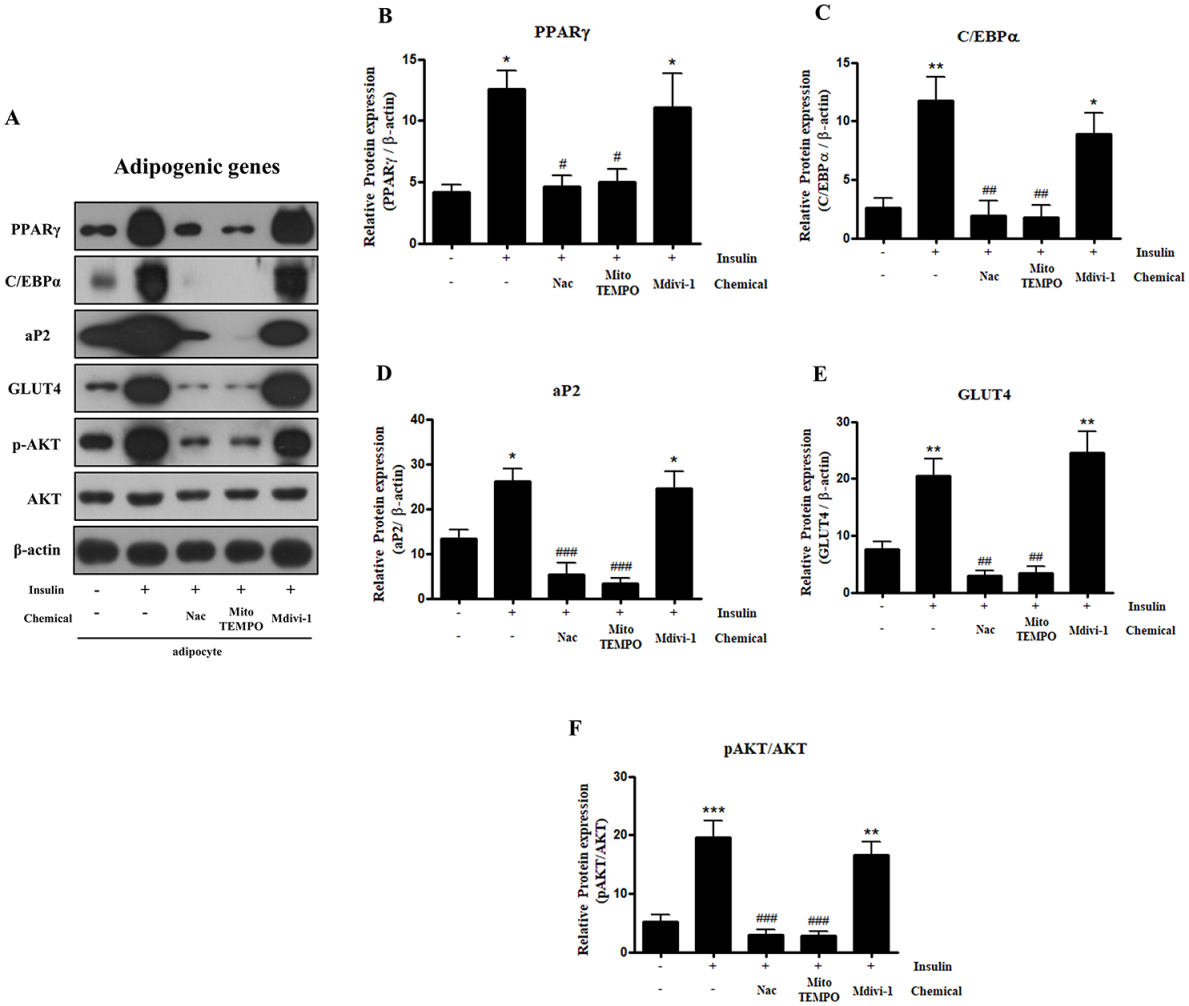
Effect of N-acetyl cysteine (Nac), Mito-TEMPO, and Mdivi-1 on the expression of adipogenic related genes in 3T3-L1 cells after differentiation. 3T3-L1 adipocytes were pretreated with 10 mM Nac, 200 μM Mito-TEMPO, or 50 μM Mdivi-1 for 30 min and then with insulin during adipogenesis. Cells were lysed and proteins were subjected to western blot analysis with the indicated antibodies: adipogenic genes (PPARγ, C/EBPα, p-AKT, AKT, GLUT4, and aP2). Data in the bar graphs represent the means ± SEM of three independent experiments. *p < 0.05, **p < 0.01, ***p < 0.001, compared with untreated cells. ^#^p < 0.05, ^##^p < 0.01, ^###^p < 0.001 compared with insulin-treated cells.

After observing the above data, we investigated the changes in adipogenic gene expression. Protein expression levels of the genes PPARγ, C/EBPα, and aP2 were significantly increased following insulin treatment and dramatically decreased following treatment with Nac or Mito-TEMPO. However, the expression of adipogenic genes was not decreased after Mdivi-1 treatment (Fig 3A–3D). Levels of phosphorylated AKT and GLUT4, a downstream effector of AKT, were significantly increased following treatment with insulin, and these decreased following treatment with Nac or Mito-TEMPO. However, the expression of phosphorylated Akt and GLUT4 was not decreased following treatment with Mdivi-1 (Fig 3A, 3E and 3F). These results indicate that antioxidants such as Nac or Mito-TEMPO inhibit lipid accumulation in state of adipocyte differentiation through alleviating the expression levels of adipogenic proteins. However, Mdivi-1 does not inhibit the expression levels of adipogenic proteins.

### Insulin treatment during adipocyte differentiation results in increased mitochondrial fission that is attenuated by Nac, Mito-TEMPO, and Mdivi-1

**Fig 4:**
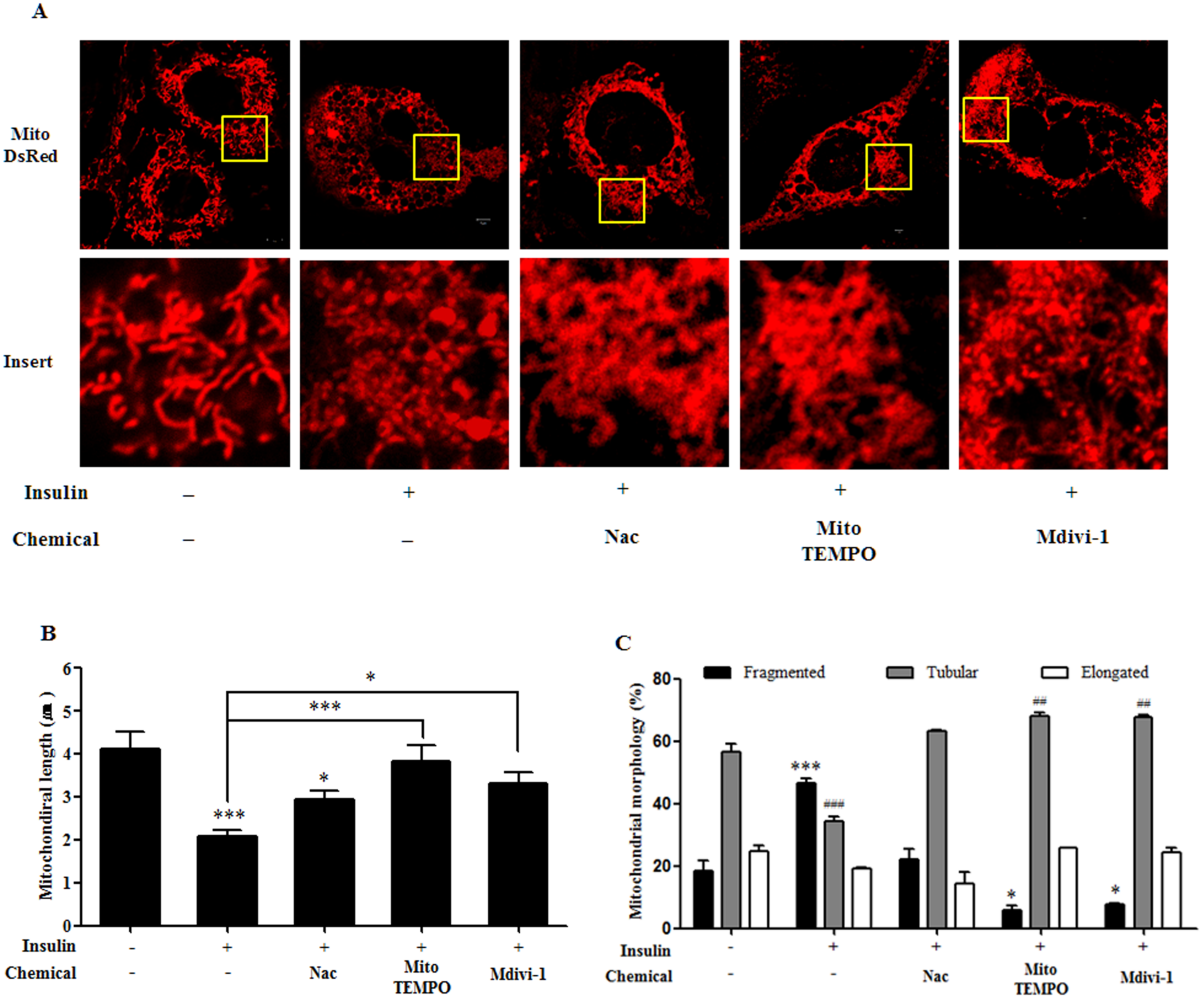
Effect of N-acetyl cysteine (Nac), Mito-TEMPO, and Mdivi-1 treatment on mitochondrial morphology in 3T3-L1 cells after differentiation. (A) DsRed2-Mito-expressing 3T3-L1 cells treated with 1 μg/mL insulin for 6 days with or without 10 mM Nac, 200 μM Mito-TEMPO, or 50 μM Mdivi-1 after MDI treatment for 48 h. Mitochondrial morphology was determined by confocal microscopy. (B) Graph showing average mitochondrial length. (C) Graph indicating mitochondrial morphology. Data in the bar graphs represent the means ± SEM of three independent experiments. *p < 0.05, **p < 0.01, ***p < 0.001 compared with untreated or insulin-treated cells. ^##^p < 0.01, ^###^p < 0.001 compared with untreated cells.

**Fig 5:**
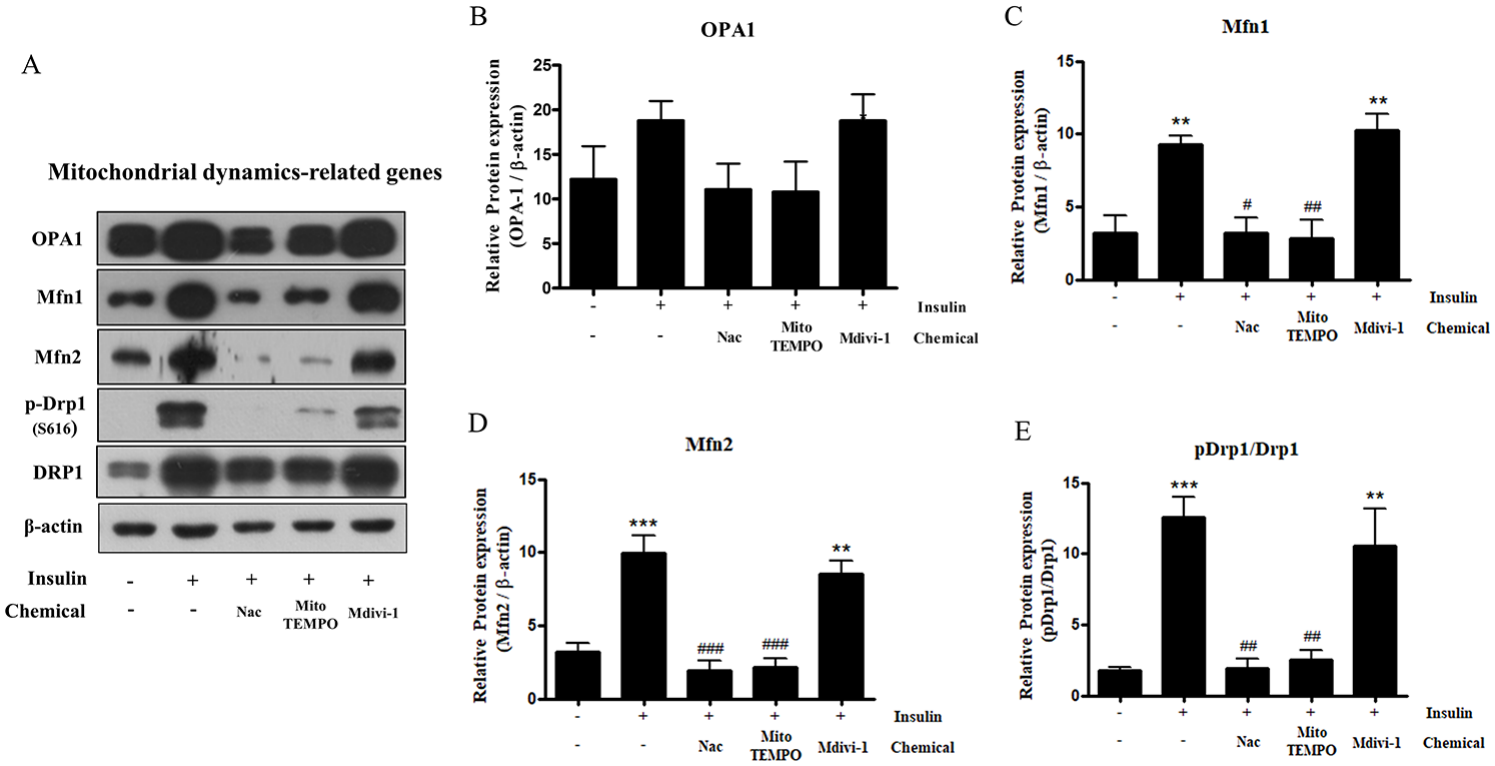
Effect of N-acetyl cysteine (Nac), Mito-TEMPO, and Mdivi-1 on mitochondrial dynamics-related genes in 3T3-L1 cells after differentiation. 3T3-L1 adipocytes were pretreated with 10 mM Nac, 200 μM Mito-TEMPO, or 50 μM Mdivi-1 for 30 min and then with insulin during adipogenesis. Cells were lysed and proteins were subjected to western blot analysis with the indicated antibodies: mitochondrial dynamics-related genes (OPA1, Mfn1, Mfn2, p-Drp1 (Ser616), and Drp1). Data in the bar graphs represent the means ± SEM of three independent experiments. **p < 0.01, ***p < 0.001, compared with untreated cells. ^#^p < 0.05, ^##^p < 0.01, ^###^p < 0.001 compared with insulin-treated cells.

We investigate whether Nac, Mito-TEMPO, and Mdivi-1 treatment affects mitochondrial morphology. To investigate mitochondrial morphology, we stably expressed a mitochondria-targeting DsRed2 (DsRed2-Mito) plasmid in 3T3-L1 cells and observed the morphological changes in mitochondria of mature adipocytes by confocal microscopy. Insulin-induced mature adipocytes showed increased formation of punctate mitochondria and mitochondrial fragmentation (Fig 4A). The average length of mitochondria significantly decreased in insulin-induced mature adipocytes (Fig 4B). The number of fragmented mitochondria significantly increased, and the number of tubular mitochondria considerably decreased compared to the untreated 3T3-L1 cells, although numbers of elongated mitochondria were unchanged (Fig 4C). However, treatment with Nac, Mito-TEMPO, and Mdivi-1 resulted in a significant decrease in fragmented mitochondria (Fig 4A). The average length of mitochondria significantly increased following treatment with Nac, Mito-TEMPO, and Mdivi-1, and the number of fragmented and tubular mitochondria clearly reversed compared with the insulin-treated mature adipocytes, although treatment with Mdivi-1 caused a slight change (Fig 4B and 4C). Accordingly, we investigated the protein expression of mitochondrial dynamics genes in 3T3-L1 cells after adipocyte differentiation. The results showed that levels of OPA-1, Mfn1, and Mfn2, associated with mitochondrial fusion, were increased in mature adipocytes after insulin treatment (Fig 5A–5D). Furthermore, levels of Drp1 phosphorylated at serine 616 increased in mature adipocytes, indicating mitochondrial fission (Fig 5A and 5E). Interestingly, protein expression of these genes was significantly decreased by Nac and mito-TEMPO, and phosphorylated Drp1 was slightly decreased compared with insulin-only treatment (Fig 5). Thus, our results clearly show that antioxidants and a Drp-1 inhibitor prevent mitochondrial fission and increase mitochondrial dynamics during insulin-induced adipocyte differentiation.

### Insulin-stimulated ROS production is inhibited by Nac and Mito-TEMPO, but not by Mdivi-1

**Fig 6:**
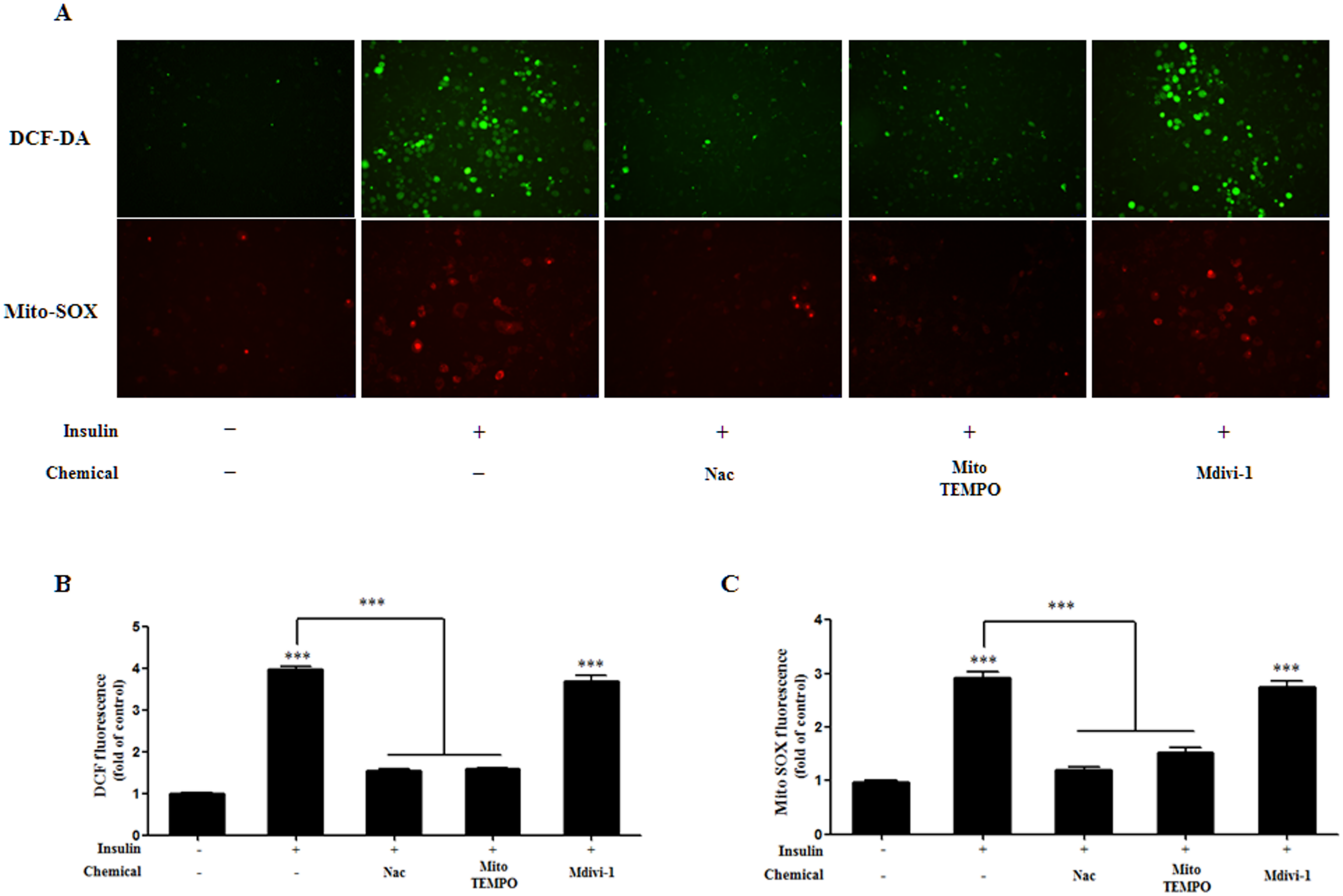
Effect of N-acetyl cysteine (Nac), Mito-TEMPO, and Mdivi-1 treatment on intracellular and mitochondrial ROS production in 3T3-L1 cells after differentiation. (A) 3T3-L1 adipocytes were pretreated with 10 mM Nac, 200 μM Mito-TEMPO, or 50 μM Mdivi-1 for 30 min and then with insulin during adipogenesis. After 8 days of the initiation of differentiation, cells were used in CM-H_2_DCF-DA (A, B) and Mito-SOX (A, C) stained assays. Data in the bar graphs represent the means ± SEM of three independent experiments. ***p < 0.001 compared with untreated or insulin-treated cells.

To investigate levels of ROS production, we cultured 3T3-L1 cells with Nac, Mito-TEMPO, or Mdivi-1 during adipocyte differentiation. As shown in our ROS staining results, cytosolic ROS levels were increased following insulin treatment using CM-H2DCF-DA, and treatments with Nac or Mito-TEMPO resulted in a significant decrease in cytosolic ROS production. With Mito-SOX staining, mitochondrial ROS levels were also increased following insulin treatment, and treatment with Nac or Mito-TEMPO resulted in a significant decrease in mitochondrial ROS production. However, treatment with Mdivi-1 resulted in no decrease of either cytosolic or mitochondrial ROS (Fig 6A–6C). Our results show that Nac and Mito-TEMPO treatment results in decreased insulin-induced ROS production; however, Mdivi-1 treatment does not inhibit ROS production.

### Expression of insulin-induced antioxidant gene proteins is inhibited by Nac and Mito-TEMPO, but not by Mdivi-1 treatment

**Fig 7:**
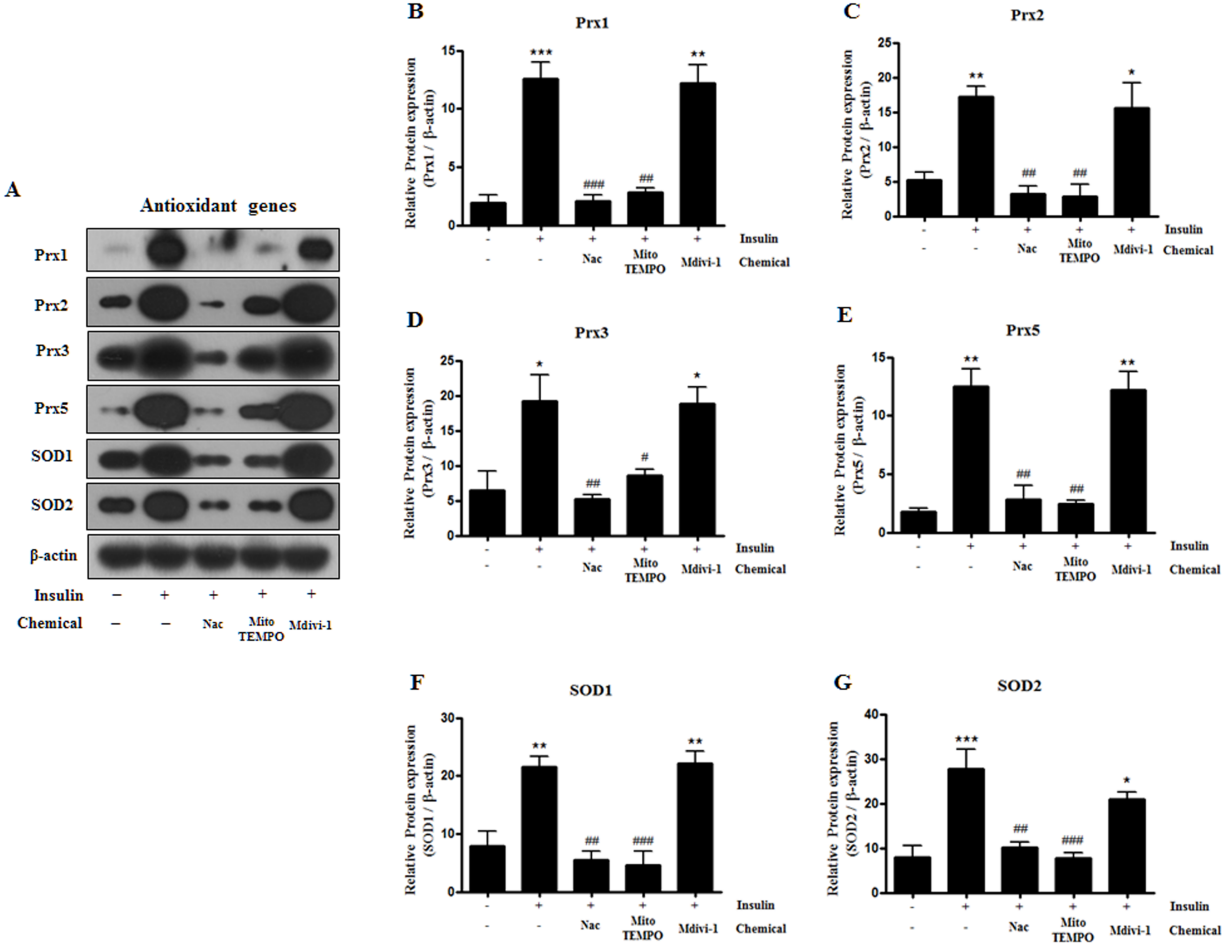
Effect of N-acetyl cysteine (Nac), Mito-TEMPO, and Mdivi-1 treatment on antioxidant related genes in 3T3-L1 cells after differentiation. (A) 3T3-L1 adipocytes were pretreated with 10 mM Nac, 200 μM Mito-TEMPO, or 50 μM Mdivi-1 for 30 min and then with insulin during adipogenesis. Cells were lysed and proteins were subjected to Western blot analysis analysis with the indicated antibodies; antioxidant genes (Prx1, Prx2, Prx3, Prx5, SOD1, and SOD2). Data in the bar graph represent the means ± SEM of three independent experiments. * p< 0.05, ** p< 0.01, *** p< 0.001, compared with not treated cell. # p< 0.05, ## p< 0.01, ### p< 0.001, compared with insulin-treated cell.

After observing the above data, we also investigated the altered expression of antioxidant genes. Protein expression levels of Prx1, Prx2, Prx3, Prx5, SOD1, and SOD2 were significantly increased following insulin treatment, and highly decreased following treatment with Nac or Mito-TEMPO. However, the expression of these antioxidant genes was not decreased after Mdivi-1 treatment (Fig 7A–7G). These results indicate that ROS scavenger chemicals inhibit antioxidant gene expression during adipocyte differentiation by decreasing levels of ROS production. However, Mdivi-1 does not inhibit the expression levels of antioxidant proteins.

## Discussion

Adipogenesis is a differentiation process in which preadipocytes become mature adipocyte cells; it is a complex and programmed process involving coordinated changes in morphology, hormone sensitivity, and gene expression [5]. In state of adipocyte differentiation, the cells require high level of energy, which elevates mitochondrial metabolism. This result shows that elevated ROS generation is essential for both the expression of key adipogenesis-related genes and transition from preadipocyte to mature adipocyte [13, 15, 19, 25]. Mitochondria are highly dynamic organelles and undergo continuous fission and fusion events in physiological situations [16, 26]. A previous study revealed that mitochondrial biogenesis and remodeling are essential for adipose differentiation and are influenced by insulin [27]. In preadipocytes, mitochondria are elongated and well organized. In contrast, in mature adipocytes, they are fragmented, punctate, and redistributed around lipid droplets [28]. Mitochondrial fission and fusion have an influence on lipid accumulation in adipocytes [29]. The fragmentation of mitochondrial tubules is associated with enhanced production of ROS [30]. However, the correlation between insulin-induced ROS production and mitochondrial remodeling networks on lipid accumulation during adipocyte differentiation remains unclear.

Several studies involving adipocytes have shown that insulin is a potent adipogenic hormone that induces a series of transcription factors during differentiation of pre-adipocytes into mature adipocytes [18, 31]. Insulin also stimulates ROS generation through mitochondrial metabolism and Nox4 activation, resulting in promotion of adipocyte differentiation [15, 25]. This Nox4-induced ROS generation is concerned with both enhanced insulin receptor autophosphorylation and the inhibition of cellular protein tyrosine phosphatase (PTP) activity by oxidation, resulting in elevation of the insulin signaling pathway [32]. ROS generation by Nox4 mediates the insulin signaling cascade in adipocytes. Treatment of H_2_O_2_ in 3T3-L1 cells induced the activation of the PI3K/AKT signaling cascade, increased basal GLUT4 vesicle translocation into plasma membrane, and ultimately increased cellular glucose uptake [33]. Among adipogenesis-related transcription factors, C/EBPα and PPARγ are key factors of white adipocyte differentiation [7, 34]. C/EBP β is induced by increased production of ROS in early-stage adipocyte differentiation, and then increased expression of PPARγ and aP2 accelerates mature adipocyte differentiation [35–37]. In the current study, we investigated whether the expression of adipogenic genes, antioxidant enzymes, and ROS generation increased after insulin treatment. As expected, we found that levels of adipogenic protein expression gradually increased during insulin-induced adipocyte differentiation (Fig 1A). Furthermore, the adipogenic gene levels were significantly reduced by the antioxidant Nac (Fig 3). These results corroborated those of our previous study [24]. Therefore, insulin-induced adipogenic-related genes mediate ROS levels in differentiated 3T3-L1 cells.

During adipogenesis, mitochondria adapt to the increased numbers of lipid droplets by fragmentation, network redistribution, and uncoupling of respiration [22]. Mitochondrial fragmentation is regulated by Drp1, and fusion is controlled by Mfn1, Mfn1, and Opa1 [38]. Furthermore, Drp1 activity is regulated by phosphorylation. Therefore, under conditions such as oxidative stress and high glucose stimulation, phosphorylation of Drp1 (at Ser616) increases [39, 40]. In this study, our data show that the protein expression of Drp1, Mfn1, Mfn2, and Opa1 increased during 3T3-L1 adipocyte differentiation (Fig 1B); these results agree with those of a previous study [22]. Accordingly, mitochondrial dynamics is required for the accumulation of lipid droplets.

The correlation between oxidative stress and mitochondrial fission has emerged in numerous studies. Oxidative stress is thought to be a regulator of mitochondrial fragmentation, and inhibition of ROS effectively represses mitochondrial fragmentation [23, 41–44]. In addition, differentiation is a highly energy-demanding status, and mitochondrial ROS are required to initiate adipocyte differentiation [19, 25]. In this study, we investigated mitochondrial morphological changes after insulin-induced ROS generation in mature adipocytes. First, we chose to investigate lipid accumulation and the effect of Mito-TEMPO on mitochondrial ROS production in insulin-induced mature adipocytes. Our results show that Mito-TEMPO not only reduces mitochondrial ROS, but also represses lipid accumulation (Figs 2 and 6). Furthermore, increased mitochondrial length and numbers of tubular mitochondria, as well as reduced numbers of fragmented mitochondria, resulted from treatment with Mito-TEMPO (Fig 4). Next, a chemical inhibitor of Drp1 have identified Dynasore and Mdivi-1 through previous study. Dynasore inhibits GTPase activity by binding of Dynamin GTPase domain, which is included on both Dynamins and Drp1, while Mdivi-1 only inhibits Drp1 assembly on outer membrane of mitochondria [45, 46]. Thus, we chose Mdivi-1 to confirm the correlation between Drp1-mediated mitochondrial fission and adipocyte differentiation. We investigated whether Mdivi-1, a specific Drp1 inhibitor, could prevent lipid accumulation in insulin-induced mature adipocytes. A previous study showed that inhibition of Drp1 or overexpression of Mfn2, a mitochondrial fusion marker, resulted in decreased TG accumulation in 3T3-L1 adipocytes [29]. Unexpectedly, our data showed that Mdivi-1 did not inhibit adipogenic gene expression and lipid accumulation in mature adipocytes (Figs 2 and 3), and mitochondrial ROS was also not inhibited by Mdivi-1 (Fig 6). However, although the protein expression of Drp1 (Ser616) decreased only slightly, treatment with Mdivi-1 resulted in significantly increased mitochondrial length and numbers of tubular mitochondria, and reduced numbers of fragmented mitochondria than insulin-only treated mature adipocytes (Figs 4 and 5). According to previous studies, mitochondrial ROS regulates C/EBPα and PPARγ, which can lead to adipocyte differentiation [47–49]. In the current study, our results indicate that mitochondrial ROS levels are not reduced by Mdivi-1, and it does not inhibit lipid accumulation. Therefore, although emerging data suggest that mitochondrial fission is inhibited by Mdivi-1, lipid accumulation is more influenced by ROS generation.

Studies in humans have revealed that adipose tissue from obese patients is associated with increased systemic oxidative stress, which might indicate a target for new therapies for obesity-related metabolic syndrome [50]. Our previous study also suggests that antioxidant materials could potentially be used to prevent obesity [24]. Accordingly, we investigated the levels of antioxidants such as Prxs and SOD. The results showed that these levels gradually increased during the transition from pre-adipocyte to mature adipocyte, which corresponded with increased ROS generation (Fig 1), and these increases were reversed by antioxidant treatment with Nac and Mito-TEMPO (Fig 7). However, levels of Prxs and SOD were not decreased by Mdivi-1 due to the lack of inhibition of ROS generation (Fig 7). We thought that maybe ROS are an upstream regulator of mitochondrial fragmentation. Meanwhile, Prx1, Prx3, and Prx5 play important roles in maintaining normal adipocyte characteristics. Furthermore, it have been reported that SOD expression as well as activity rose during adipocyte differentiation in 3T3-L1 [13, 51]; therefore, redox regulation by antioxidants is required for adipocyte differentiation. However, the precise role of antioxidants in the regulation of mitochondrial dynamics during adipocyte differentiation remains unclear. Furthermore, there are still necessary to study about the effect of C/EBP β gene expression and mitochondria fission with or without antioxidant enzyme on early stage adipocyte differentiation. Thus, we will be investigate the effect of C/EBP β gene expression and mitochondria fission when it ectopic overexpression of the antioxidant gene such as peroxiredoxin 2 or 5 during induction or early stage of differentiation in 3T3L1 and Primary pre-adipocyte from mice white adipose tissue.

In conclusion, our results demonstrate that the regulation of adipogenic genes and altered mature adipocytes are influenced by ROS generation, and merely suppressing mitochondrial fragmentation does not inhibit lipid accumulation during adipocyte differentiation. These findings provide a fundamental basis for understanding the correlation between ROS and mitochondrial dynamics in adipocyte differentiation, which may lead to the development of ROS-based therapeutic strategies for the suppression of obesity.
